# Fibrinogen–Albumin-Ratio is an independent predictor of thromboembolic complications in patients undergoing VA-ECMO

**DOI:** 10.1038/s41598-021-95689-x

**Published:** 2021-08-17

**Authors:** Sebastian Roth, Catrin Jansen, René M’Pembele, Alexandra Stroda, Udo Boeken, Payam Akhyari, Artur Lichtenberg, Markus W. Hollmann, Ragnar Huhn, Giovanna Lurati Buse, Hug Aubin

**Affiliations:** 1 Department of Anesthesiology, Medical Faculty and University Hospital Duesseldorf, Heinrich-Heine-University Duesseldorf, Moorenstr. 5, 40225 Duesseldorf, Germany; 2 Department of Cardiac Surgery, Medical Faculty and University Hospital Duesseldorf, Heinrich-Heine-University Duesseldorf, Moorenstr. 5, 40225 Duesseldorf, Germany; 3 Department of Anesthesiology, Amsterdam University Medical Center (AUMC), Location AMC, Meibergdreef 9, 1105 AZ Amsterdam, The Netherlands

**Keywords:** Cardiac device therapy, Predictive markers

## Abstract

Veno-arterial extracorporeal membrane oxygenation (VA-ECMO) supports patients suffering from refractory cardiogenic shock. Thromboembolic complications (TeC) are common in VA-ECMO patients and are associated with increased morbidity and mortality. Valid markers to predict TeC in VA-ECMO patients are lacking. The present study investigated the predictive value of baseline Fibrinogen–Albumin-Ratio (FAR) for in-hospital TeC in patients undergoing VA-ECMO. This retrospective cohort study included patients who underwent VA-ECMO therapy due to cardiogenic shock at the University Hospital Duesseldorf, Germany between 2011 and 2018. Main exposure was baseline FAR measured at initiation of VA-ECMO therapy. The primary endpoint was the in-hospital incidence of TeC. In total, 344 patients were included into analysis (74.7% male, mean age 59 ± 14 years). The in-hospital incidence of TeC was 34%. Receiver operating characteristics (ROC) curve of FAR for in-hospital TeC revealed an area under the curve of 0.67 [95% confidence interval (CI) 0.61–0.74]. Youden index determined a cutoff of 130 for baseline FAR. Multivariate logistic regression revealed an adjusted odds-ratio of 3.72 [95% CI 2.26–6.14] for the association between FAR and TeC. Baseline FAR is independently associated with in-hospital TeC in patients undergoing VA-ECMO. Thus, FAR might contribute to the prediction of TeC in this cohort.

## Introduction

Venoarterial extracorporeal membrane oxygenation (VA-ECMO) is used to temporarily support the cardiac cycle and gas exchange in patients with acute cardiorespiratory failure^1–3^. Despite of continuous improvements in oxygenators and pump technologies, VA-ECMO therapy is still associated with a high rate of complications^4–6^. Previous studies state a mortality rate between 40 and 60%^7,8^. The incidence of thromboembolic complications (TeC) such as ischemic stroke, cannula-associated deep vein thrombosis or arterial thromboembolism is estimated at 33%, 41% and 14% respectively^9^. These data show clearly that further improvement of VA-ECMO therapy is warranted. One approach is to identify prognostic biomarkers, for example to predict thromboembolic events in advance^10^. This could possibly improve the outcome due to early identification of therapeutic measures and potential treatment targets. In addition, the use of valid biomarkers could help to understand which patients could really benefit from VA-ECMO.

The Fibrinogen–Albumin-Ratio (FAR) has been suggested as an indicator for disease severity during prothrombotic conditions^11–13^. Fibrinogen and Albumin both have effects on blood clotting. While Fibrinogen is a clotting factor that elevates the aggregation of thrombocytes^14^, Albumin plays a role in inhibiting the function of thrombocytes and thrombus formation^15,16^. So far, data on the prognostic value of FAR for patients with VA-ECMO are scarce. A retrospective cohort study revealed that an elevated FAR within the first 24 h after initializing VA-ECMO therapy was associated with a higher risk of ischemic stroke^17^. To our best knowledge, the association between FAR and TeC in general has not been investigated yet. Therefore, we aimed to determine whether early FAR is associated with in-hospital TeC in patients undergoing VA-ECMO therapy.

## Methods

This retrospective, single-center cohort study was conducted according to the guidelines for good clinical practice (GCP) and the declaration of Helsinki. The study was approved by the ethical committee of the Heinrich-Heine-University, Duesseldorf, Germany (reference number 5141R). All patients gave written informed consent to be registered in a dedicated database. This manuscript follows the STROBE reporting guidelines for retrospective cohort studies.

### Participants

The present study included all patients who underwent extracorporeal life support (ECLS) at the University Hospital Duesseldorf, Germany between 2011 and 2018 due to refractory cardiogenic shock. Exclusion criteria were missing data regarding the primary endpoint, incomplete medical records so that documentation of TeC was not possible, age < 18 years and the use of veno-venous ECMO. Anticoagulation was performed with unfractionated heparin according to the local standard or with argatroban, if appropriate. Anticoagulation monitoring was based on activated partial thromboplastin time (aPTT) or anti-factor Xa-activity.

### Definition and assessment of main exposure

Main exposure was FAR on the day of initiation of VA-ECMO therapy. Measurements of Fibrinogen and Albumin values were performed in the central laboratory of the University Hospital Duesseldorf. The FAR was calculated by dividing Fibrinogen to Albumin^12,17^. Furthermore, baseline Fibrinogen and baseline Albumin were investigated alone to see if the ratio of both values is superior to the single values. In an additional analysis, FAR on day five was analysed as alternative exposure. The decision to choose these two time points for analysis was based on the rationale that VA-ECMO initiation itself might be associated with TeC, for example due to cannula associated thrombosis. Hence, the predictive value of FAR before or after start of VA-ECMO therapy might be different. Day five was chosen based on local experiences suggesting that this might be a typical point of time where clinicians often have to decide about prognosis and whether therapy should be continued or not.

### Outcome assessment

The primary endpoint of this study was the incidence of in-hospital TeC. TeC were defined as a composite of non-fatal arterial thrombosis or embolism, non-fatal venous thrombosis or embolism, non-fatal ischemic stroke, non-fatal myocardial infarction, or thromboembolic vascular mortality^18^. Arterial and venous thromboembolisms were defined as any new and symptomatic non-cardiac and non-cerebral arterial or venous thrombosis or embolism not causing death. Non-fatal myocardial infarction was defined according to the fourth universal definition of myocardial infarction^19^. Non-fatal ischemic stroke was defined according to the guidelines by the American Stroke Association^20^. Thromboembolic vascular mortality was defined as any TeC causing death. Data on TeC were extracted from electronic medical charts by personnel trained in the study definitions. TeC was confirmed when there was a clearly documented diagnosis that was approved by a physician specialized in intensive care medicine. Plausibility checks were done whenever further source documents were available.

### Sample size

Due to the nature of this retrospective exploratory data analysis, we did not conduct formal sample size calculation. However, based on the current literature, we expected TeC in approximately 30% of patients^8^. With an estimated study sample of 350 patients, we expected approximately 105 thromboembolic events. This allowed to include up to 10 predefined co-variables for multivariable adjustment (see “Statistical analysis”). As 117 events could be observed in this study, we were able to add two further covariates (= 12 covariates in total) to a separate analysis that was conducted post factum during review process.

### Statistical analysis

Patient characteristics are presented as absolute values with corresponding percentages for categorical data or as mean ± standard deviation for continuous data, as appropriate. Shapiro–Wilks test was used to test for normal distribution of data. Fisher exact test and t-tests were used to test for differences between categorical and dichotomous data. Discrimination of baseline FAR for in-hospital TeC was analyzed by receiver operating characteristics (ROC) curve and the “area under the curve” (AUC). ROC analysis was also done for Fibrinogen and Albumin alone. De Long-Test was performed to compare ROC curves. A cutoff value for FAR was determined by Youden Index. Multivariable logistic regression analysis was used to assess the independent association (Odds ratio (OR); 95% confidence interval (CI)) between elevated FAR and in-hospital TeC after adjustment by the following predefined covariables (forced entry): age, sex, chronic coronary syndrome, history of ischemic stroke, history of pulmonary embolism, arterial hypertension, diabetes mellitus, days of VA-ECMO therapy, continuous veno-venous hemodialysis treatment during hospitalization. The choice of covariates was based on literature research^2,3,21–23^ and/or clinical experiences so that covariates were included if an association with TeC seemed possible. Baseline quick and baseline activated partial thromboplastin time (aPTT) could be added to an additional post factum logistic regression model. Model calibration was assessed using Hosmer–Lemeshow-Test. Net reclassification index (NRI) and integrated discrimination index (IDI) were calculated for FAR and for Fibrinogen and Albumin alone. For all statistical tests, *p* < 0.05 was considered significant. Analyses were performed with IBM SPSS Statistics version 26 (IBM, Armonk, New York, United States) and GraphPad-Prism^©^ statistical software version 6 (GraphPad software Inc, San Diego, California, United States).

### Ethics approval and consent to participate

The study was approved by the ethical committee of the Heinrich-Heine-University, Duesseldorf, Germany (reference number 5141R). All patients gave written informed consent to be registered in a dedicated database.

## Results

### Study cohort and baseline characteristics

**Figure 1:**
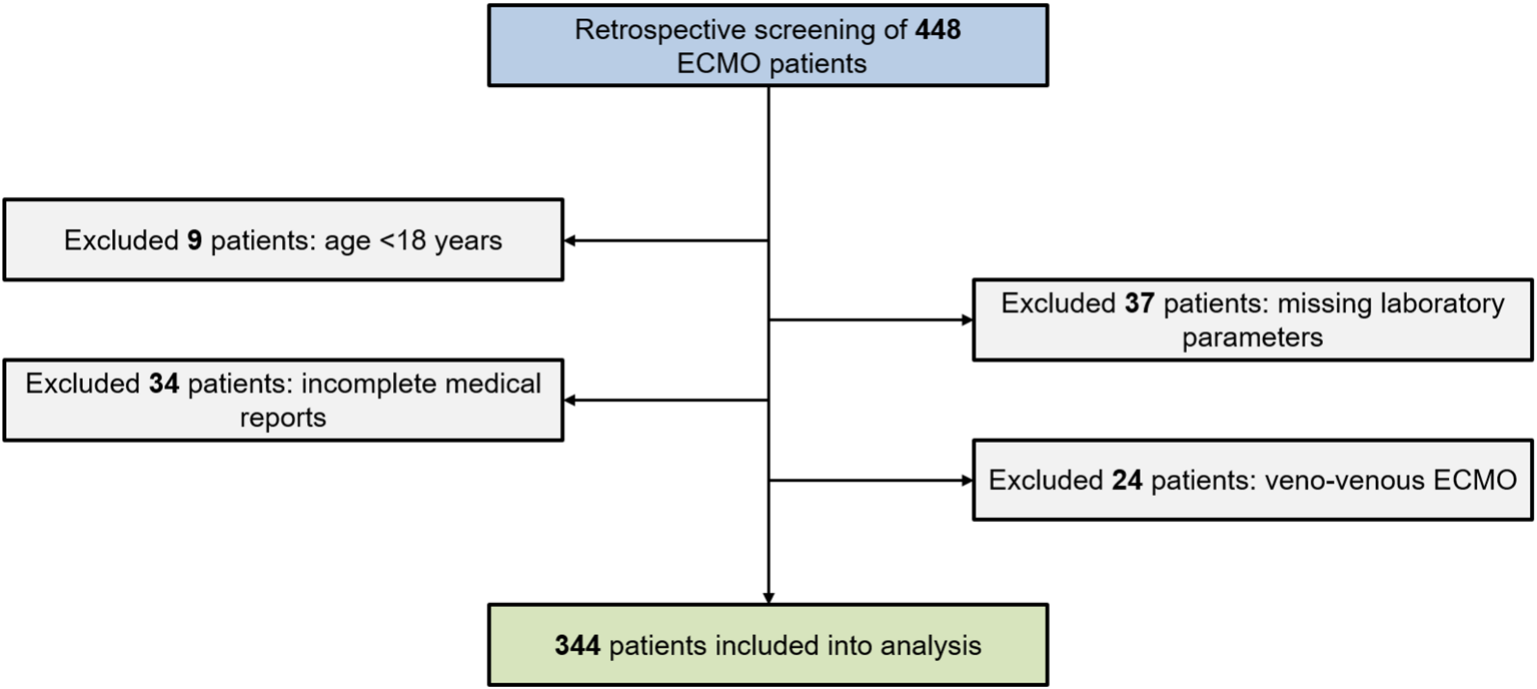
Study flow chart.

**Figure 2:**
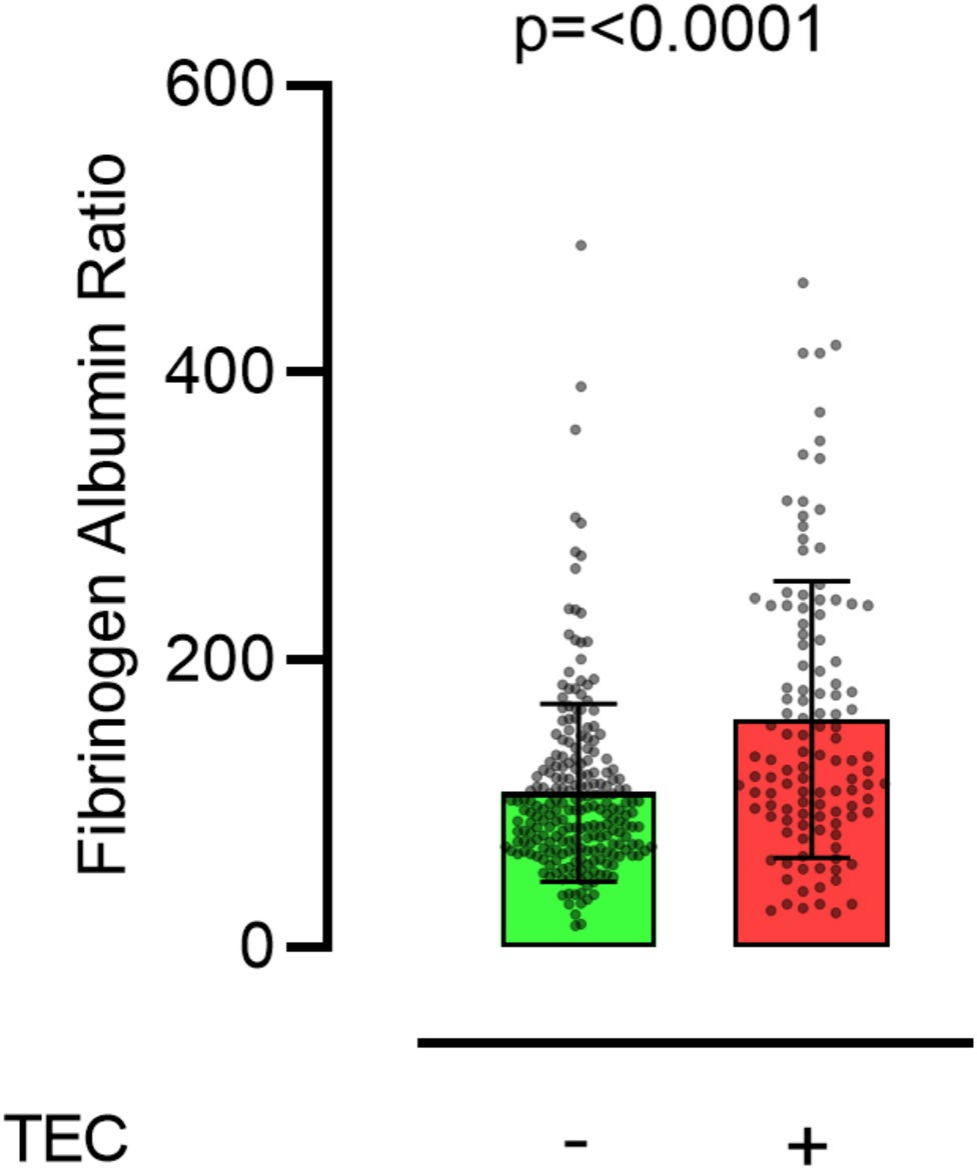
Box plot shows that Fibrinogen–Albumin-Ratio levels in patients with or without thromboembolic complication (TEC) are significantly different.

The study flow chart is shown in Fig. 1. Of the included 344 patients, 257 (74.7%) were male, the mean age was 59 ± 14. Table 1 reports detailed patients characteristics of the whole cohort and by primary outcome. In total, 117 patients (34%) had a TeC during their hospital stay. The most common TeCs were arterial thromboembolic events (63 patients = 18.3%) and ischemic stroke (40 patients = 11.6%). Overall in-hospital mortality was 58.1% (200/344). Patients with TeC during hospitalization had a significantly higher FAR (158 ± 96) than patients without TeC (108 ± 62) (see Fig. 2). Table 2 summarizes characteristics by FAR above and below the cut-off established by Youden Index. Patients with FAR < 130 had significantly more major bleedings (122 (52.8%) versus 44 (38.9%)) and a higher rate of acute kidney injury requiring renal replacement therapy (132 (57.1%) versus 78 (69.0%)). In addition, patients with FAR below the calculated cutoff had significantly fewer non-fatal myocardial infarctions (3 (1.3%) versus 8 (7.1%)) and other non-fatal arterial thromboembolisms (29 (12.6%) versus 34 (30.1%)). Furthermore, patients with FAR < 130 had a significantly lower baseline quick value (45 ± 20% versus 52 ± 20%).

**Table 1:** Baseline characteristics of the whole cohort and in patients without and with TeC.

	All VA-ECMO patients (N = 344)	Patients without TEC (N = 227)	Patients with TEC (N = 117)	p-value^a^
**Baseline characteristics**				
Male sex no. (%)	257 (74.7)	167 (73.6)	90 (76.9)	0.516
Age (years)	59 ± 14	58.7 ± 14.9	58.8 ± 13.7	0.930
Duration of ECMO (days)	7.6 ± 5.9	6.7 ± 5.2	9.3 ± 6.9	< 0.0001
**Comorbidities no. (%)**				
Arterial hypertension	111 (32.3)	73 (32.2)	38 (32.5)	0.999
Diabetes	72 (20.9)	46 (20.3)	26 (22.2)	0.677
Chronic coronary syndrome	176 (51.2)	123 (54.2)	53 (45.3)	0.139
Peripheral artery disease	37 (10.8)	20 (8.8)	17 (14.5)	0.141
Prior MI	162 (47.1)	99 (43.6)	63 (53.8)	0.087
Prior stroke	22 (6.4)	14 (6.2)	8 (6.8)	0.819
Prior pulmonary embolism	14 (4.1)	7 (3.1)	7 (6.0)	0.250
**Clinical endpoints no. (%)**				
In hospital death	200 (58.1)	133 (58.6)	67 (57.3)	0.819
Major bleeding	166 (48.3)	115 (50.7)	51 (43.6)	0.255
AKI with CVVHD	210 (61.0)	127 (55.9)	83 (70.9)	0.007
**Thromboembolic complications no. (%)**	117 (34.0)			
MI	11 (3.2)	0 (0)	11 (9.4)	< 0.0001
Stroke	40 (11.6)	0 (0)	40 (34.2)	< 0.0001
Art. thromboembolism	63 (18.3)	0 (0)	63 (53.8)	< 0.0001
Ven. thromboembolism	11 (3.2)	0 (0)	11 (9.4)	< 0.0001
Vascular death	3 (0.9)	0 (0)	3 (2.6)	0.039
**Laboratory parameters at baseline**				
Creatinine (mg/dl)	1.9 ± 1.6	1.9 ± 1.5	1.9 ± 1.6	0.944
Leukocytes (× 1000/µl)	14.5 ± 7.5	14.7 ± 7.6	14.1 ± 7.3	0.492
Hemoglobin (g/dl)	10.7 ± 2.3	10.6 ± 2.3	10.8 ± 2.4	0.377
Hematocrite (%)	33 ± 17	33.3 ± 20.6	32.5 ± 7.5	0.688
Thrombocytes (× 1000/µl)	174 ± 97	171 ± 90	179 ± 110	0.477
aPTT (s)	76 ± 47	77 ± 48	73 ± 46	0.510
Quick (%)	48 ± 21	47 ± 21	50 ± 20	0.151
Antithrombin III (%)	55 ± 32	58 ± 36	52 ± 20	0.184
D-Dimer	16.2 ± 21.8	15 ± 20	19 ± 25	0.221
Fibrinogen (mg/dl)	288 ± 149	269 ± 139	326 ± 162	0.001
Albumin (g/l)	2.5 ± 0.8	2.6 ± 0.8	2.3 ± 0.8	0.001
FAR	125 ± 79	108 ± 62	158 ± 96	< 0.0001

Data are presented as mean ± standard deviation or as absolute values with percentages, as appropriate.

*TEC* thromboembolic complication, *AKI* acute kidney injury, *aPTT* activated partial thromboplastin time, *CVVHD* continuous Veno-venous hemodialysis, *VA-ECMO* veno-arterial extracorporeal membrane oxygenation, *FAR* Fibrinogen–Albumin ratio, *MI* myocardial infarction.

^a^*p* value of Chi-square test or two-tailed unpaired t-test after Levene’s test for equality of variances.

**Table 2:** Baseline characteristics in patients with FAR < 130 and ≥ 130.

	Patients with FAR < 130 (N = 231)	Patients with FAR ≥ 130 (N = 113)	p-value^a^
**Baseline characteristics**			
Male sex no. (%)	169 (73.2)	88 (77.9)	0.359
Age (years)	58 ± 15	59 ± 14	0.788
Duration of ECMO (days)	7.5 ± 5.9	7.7 ± 6.0	0.777
**Comorbidities no. (%)**			
Arterial hypertension	76 (32.9)	35 (31.0)	0.806
Diabetes	49 (21.2)	23 (20.4)	0.889
Chronic coronary syndrome	121 (52.4)	55 (48.7)	0.566
Peripheral artery disease	26 (11.3)	11 (9.7)	0.715
Prior MI	109 (47.2)	53 (46.9)	0.999
Prior stroke	16 (6.9)	6 (5.3)	0.646
Prior pulmonary embolism	9 (3.9)	5 (4.4)	0.779
**Clinical endpoints no. (%)**			
In hospital death	135 (58.4)	65 (57.5)	0.908
Major bleeding	122 (52.8)	44 (38.9)	0.016
AKI with CVVHD	132 (57.1)	78 (69.0)	0.035
**Thromboembolic complications no. (%)**	56 (24.2)	61 (54.0)	< 0.0001
MI	3 (1.3)	8 (7.1)	0.007
Stroke	23 (10.0)	17 (15.0)	0.209
Art. thromboembolism	29 (12.6)	34 (30.1)	< 0.0001
Ven. thromboembolism	8 (3.5)	3 (2.7)	0.999
Vascular death	1 (0.4)	2 (1.8)	0.252
**Laboratory parameters at baseline**			
Creatinine (mg/dl)	1.9 ± 1.5	1.6 ± 1.5	0.439
Leukocytes (× 1000/µl)	14.5 ± 7.6	14.5 ± 7.3	0.965
Hemoglobin (g/dl)	10.8 ± 2.4	10.4 ± 2.0	0.122
Hematocrite (%)	33 ± 8	34 ± 28	0.424
Thrombocytes (× 1000/µl)	171 ± 93	180 ± 106	0.451
aPTT (s)	79 ± 48	69 ± 44	0.087
Quick (%)	45 ± 20	52 ± 20	0.003
Antithrombin III (%)	56 ± 36	54 ± 20	0.624
D-Dimer	17.2 ± 22.0	14.5 ± 21.2	0.299
Fibrinogen (mg/dl)	222 ± 87	423 ± 161	< 0.0001
Albumin (g/l)	2.7 ± 0.8	2.1 ± 0.6	< 0.0001
FAR	82.5 ± 26.7	212.2 ± 78.9	< 0.0001

Data are presented as mean ± standard deviation or as absolute values with percentages, as appropriate.

*AKI* acute kidney injury, *aPTT* activated partial thromboplastin time, *CVVHD* continuous Veno-venous hemodialysis, *ECMO* extracorporeal membrane oxygenation, *FAR* Fibrinogen–Albumin ratio, *MI* myocardial infarction.

^a^*p* value of Chi-square test or two-tailed unpaired t-test after Levene’s test for equality of variances.

### ROC analysis and determination of cutoff

**Figure 3:**
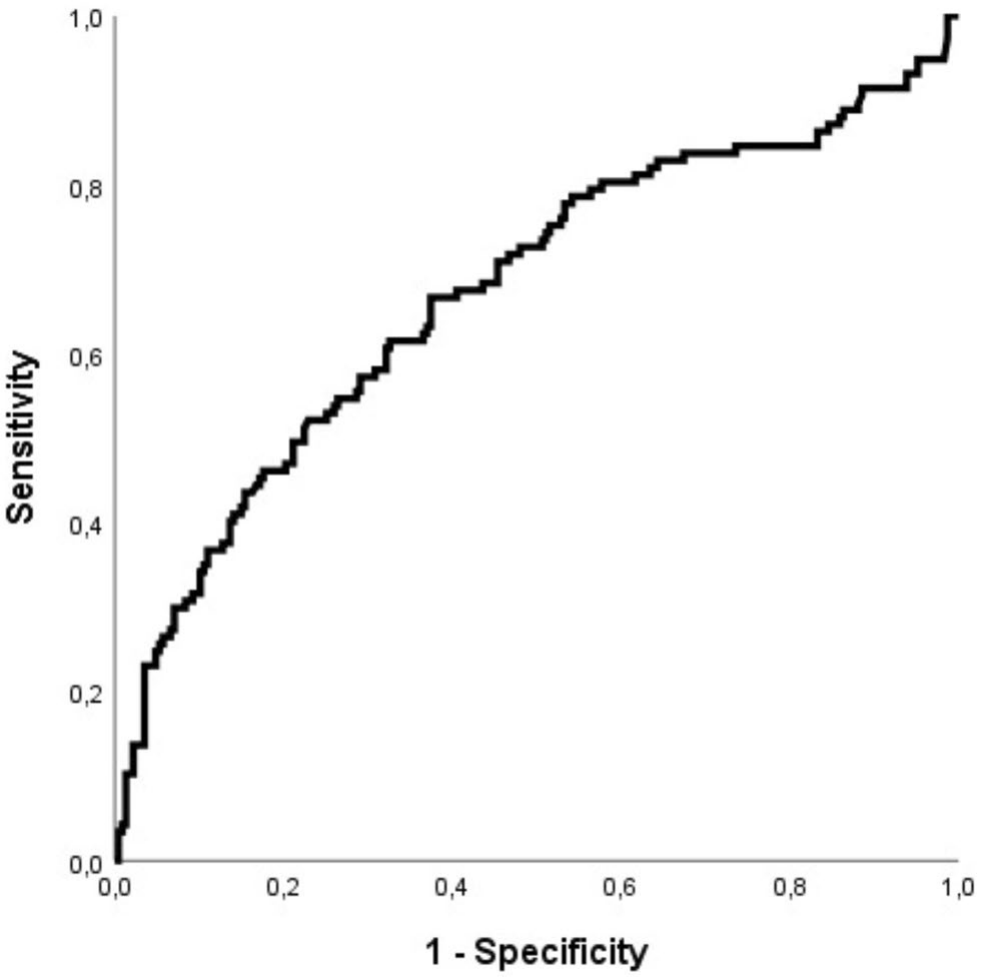
ROC curve shows moderate discrimination of baseline Fibrinogen–Albumin-Ratio for in-hospital thromboembolic complications (AUC = 0.67 [95% CI 0.61–0.74]).

ROC analysis for baseline FAR and in-hospital TeC revealed an AUC of 0.67 [95% CI 0.61–0.74; p < 0.0001] (see Fig. 3). On day five, data for 212 patients were still available. ROC analysis for FAR on day five and in-hospital TeC revealed an AUC of 0.66 [95% CI 0.57–0.76; p < 0.0001]. Youden index determined a cutoff of 130 for baseline FAR. ROC analysis for baseline Fibrinogen alone and baseline Albumin alone revealed an AUC of 0.61 [95% CI 0.54–0.67; p = 0.001] and 0.61 [95% CI 0.54–0.67; p = 0.001], respectively (see Supplementary Fig. 1). Comparison of ROC curves revealed that AUC-FAR was significantly higher than AUC-Fibrinogen (difference between areas = 0.064 [95% CI 0.018–0.111), p = 0.0066]. Difference between area of AUC-FAR and AUC-Albumin was 0.064 [95% CI − 0.002 to 0.13), p = 0.056].

### Multivariate binary logistic regression

Binary logistic regression analysis with multivariable adjustment for ten predefined co-variables revealed a significant association between baseline FAR and in-hospital TeC with an OR of 3.72 (95% CI 2.26–6.14) (see Table 3). The OR for the association between FAR on day 5 and in-hospital TeC was 5.79 [95% CI 2.41–13.89]. A post factum logistic regression model including aPTT and quick as further covariables (= 12 covariables in total) revealed no new significant or relevant findings (see Supplementary Table 1).

**Table 3:** Multivariate logistic regression for the association between baseline Fibrinogen–Albumin-Ratio and thromboembolic complications.

Covariables	Odds ratio	Lower 95% CI	Upper 95% CI	p-value
FAR	3.722	2.258	6.135	< 0.001
Sex	0.814	0.464	1.428	0.472
Age	1.004	0.986	1.022	0.667
CCS	0.661	0.393	1.114	0.120
Prior Stroke	1.066	0.392	2.895	0.900
Prior PE	2.127	0.643	7.033	0.216
Diabetes mellitus	1.255	0.669	2.354	0.479
Arterial hypertension	0.886	0.504	1.558	0.674
CVVHD	1.383	0.819	2.336	0.226
Days of VA-ECMO	1.083	1.037	1.130	< 0.001

*CCS* Chronic coronary syndrome, *CI* Confidence Interval, *CVVHD* Continous Veno-Venous Hemodialysis, *FAR* Fibrinogen–Albumin-Ratio, *PE* Pulmonary Embolism, *VA-ECMO* Veno-Arterial Extracorporeal Membrane Oxygenation.

### Net reclassification index and integrated discrimination index

The overall NRI of FAR was 40.9%. FAR-NRI for events was 26.4% [95% CI 20.8–32.7%; p < 0.0001] and FAR-NRI for non-events was 14.5% [95% CI 8.7–22.2%; p < 0.0001]. Calculation for Fibrinogen alone revealed an overall NRI of 30.1% with an Fibrinogen-NRI of 9.4% [95% CI 4.8–16.2%; p = 0.0013] for events and 20.7% [95% CI 15.6–26.6%; p < 0.0001] for non-events. Calculation for Albumin alone revealed an overall NRI of 22.1% with an Albumin-NRI of 11.1% [95% CI 6.1–18.3%; p = 0.0004] for events and 11% [95% CI 7.3–15.8%; p < 0.0001] for non-events. The IDI for FAR was 0.074 [95% CI 0.041–0.107; p < 0.0001] and IDI for Fibrinogen and Albumin alone was 0.053 [95% CI 0.025–0.081; p = 0.0002] and 0.033 [95% CI 0.012–0.056; p = 0.003], respectively.

## Discussion

The main finding of the present study is that baseline FAR is independently associated with in-hospital TeC in patients requiring VA-ECMO due to refractory cardiogenic shock. Furthermore, this study identified a cutoff of 130 for baseline FAR, which was related to a higher likelihood of TeC. The independent association between FAR and TeC was also present when FAR was measured on day 5 of VA-ECMO therapy.

### Prediction and prevention of TeC in VA-ECMO patients

One of the most important issues in terms of treating VA-ECMO patients is to understand which patients could really benefit from VA-ECMO. This decision has to be faced prior to the initiation of VA-ECMO. Once initiated, another important question in terms of prognosis and risk stratification is to decide whether VA-ECMO therapy should be continued or limited, for example if patients suffer from severe complications.

In the last decade, several scores such as the Survival after Veno-Arterial ECMO (SAVE) score^21^ have been suggested to help clinicians with these issues, but data focused on the prediction of TeC are rare. In a small retrospective cohort study with 62 patients, Trudzinski and colleagues tried to find predictors for TeC in patients undergoing veno-venous ECMO due to respiratory failure. This study found that the quality of anticoagulation and ECMO runtime predicted thromboembolic events^22^. Most other studies in this field also focused on the role of anticoagulation and the monitoring of coagulation parameters such as activated clotting time (ACT), aPTT or anti-factor Xa-activity^24,25^. Pieri et al. performed a small case–control study with a total of 20 patients to compare bivalirudin-based anticoagulation with heparin-based protocols in a population of patients treated with VV-ECMO or VA-ECMO with a target aPTT of 45–60 s^26^. The authors concluded that Bivalirudin-based anticoagulation may represent a new method of anticoagulation for reducing thromboembolic complications. A recently published study by Fisser and colleagues investigated Argatroban versus heparin in patients without heparin-induced thrombocytopenia during VV-ECMO^27^. This prospective cohort study included 465 patients and found out that Argatroban was non-inferior to Heaprin regarding bleeding and thrombosis. In summary, data regarding anticoagulation and monitoring of coagulation parameters are still inconclusive.

### FAR to predict TeC in VA-ECMO patients

Regarding pathophysiologic mechanisms behind the association of FAR and TeC, Albumin is an essential plasma protein that has been proposed to be related to inflammatory and hemostatic processes^11^. Moreover, Albumin plays a role in the inhibition of platelet activation^15^. Fibrinogen on the other hand is an indicator of a procoagulatory status and contributes to inflammation at diverse levels^12,14^. The combination of these characteristics served as a basis to hypothesize that the ratio of Fibrinogen and Albumin may predict TeC in VA-ECMO patients as this cohort is at high risk for TeC. To date—to our best knowledge—there is only one study by Acharya and colleagues that investigated the predictive value of FAR in patients undergoing VA-ECMO^17^. In a retrospective single-center cohort study, this study analysed 157 patients regarding FAR measured within the first 24 h of VA-ECMO therapy and determined its prognostic value for the incidence of in-hospital ischemic stroke^17^. This study showed a significant association between an elevated FAR (> 125) and in-hospital ischemic stroke. Our results add to these data by not only investigating the association between FAR and ischemic stroke, but with TeC in general. In addition, our data reveal a very similar cutoff for FAR (= 130) so that this cutoff seems to be suitable. Finally, our study had a larger sample size (344 vs. 157).

Referring to patient characteristics of our study, overall in-hospital mortality rate was 58.1%. This is in line with previously published data^2^. Interestingly, mortality was not influenced by TeC (see Table 1). However, it is important to mention that similar mortality rates do not automatically mean that there was no life impact. Unfortunately, we cannot provide data on more patient-centered outcomes such as “days alive and out of hospital”^28^. Another remarkable finding was that duration of VA-ECMO was significantly different between patients with or without TeC (9.3 ± 6.9 vs. 6.7 ± 5.2). This aspect is underlined by the results of multivariate analysis, which also showed that the length of VA-ECMO therapy was independently associated with TeC (OR 1.08 [95% CI 1.04–1.13]. Thus, taken together, days of VA-ECMO seem to be a relevant risk factor to develop TeC.

## Strengths and limitations

This study has several strengths. One strength of this study is that there was a high number of events (TeC: 117/344 = 34%) so that we could adjust for ten covariables in our multivariate logistic regression model (12 covariables post factum). Another strength is that all included patients were registered in a dedicated database, which ensured higher quality of our data.

This study also has limitations: first, this is a retrospective, single-center cohort study. However, baseline characteristics and in-hospital mortality rate (58.1%) were in line with previously published data^2^, suggesting that both VA-ECMO indication and outcome in our centre might be representative for larger practice. Importantly, before drawing final conclusions, the predictive value of FAR for TeC should be investigated in prospective multicenter studies. Second, no cox regression could be performed as the exact time point of TeCs was not always documented in patients’ medical records. Third, our database did not include reasons for initiation of VA-ECMO therapy. Although we know that all patients had refractory cardiogenic shock, we do not have information regarding the reason that led to cardiogenic shock.

## Conclusions

In conclusion, this study shows that baseline FAR is independently associated with in-hospital TeC in patients undergoing VA-ECMO. Therefore, FAR might be used to support the prediction of TeC in this cohort. Future studies should validate these findings with a prospective design.

## Supplementary Information

Supplementary Information.

## Data Availability

The datasets generated during and/or analysed during the current study are available from the first author on reasonable request.
