# Supplementary material for: Fibrinogen–Albumin-Ratio is an independent predictor of thromboembolic complications in patients undergoing VA-ECMO
Source: Sci Rep. 2021 Aug 17;11:16648. doi: 10.1038/s41598-021-95689-x (PMC8371004; doi:10.1038/s41598-021-95689-x)
Supplement: Supplementary file 1 — Supplementary Information. [file 41598_2021_95689_MOESM1_ESM.docx]

**Supplementary table 1:** Post factum logistic regression model for the association between

baseline Fibrinogen-Albumin-Ratio and thromboembolic complications

| Covariables | | Odds ratio | Lower 95% CI Upper 95% CI | | p-value |
| --- | --- | --- | --- | --- | --- |
|  | FAR | 3.838 | 2.285 | 6.445 | **<0.001** |
|  | Sex | 0.795 | 0.448 | 1.413 | 0.435 |
|  | Age | 1.012 | 0.993 | 1.031 | 0.230 |
|  | CCS | 0.614 | 0.362 | 1.044 | 0.072 |
|  | Prior Stroke | 1.165 | 0.427 | 3.178 | 0.766 |
|  | Prior PE | 1.769 | 0.507 | 6.168 | 0.371 |
|  | Diabetes mellitus | 1.267 | 0.666 | 2.410 | 0.470 |
|  | Arterial hypertension | 0.873 | 0.490 | 1.557 | 0.646 |
|  | CVVHD | 1.266 | 0.738 | 2.170 | 0.392 |
|  | Days of VA-ECMO | 1.089 | 1.041 | 1.139 | **<0.001** |
|  | aPTT | 1.003 | 0.996 | 1.009 | 0.410 |
|  | Quick | 1.003 | 0.989 | 1.017 | 0.688 |
|  |  |  |  |  |  |

CCS = Chronic coronary syndrome; CI = Confidence Interval; CVVHD = Continous Veno-Venous Hemodialysis; FAR = Fibrinogen-Albumin-Ratio; PE = Pulmonary Embolism; VA-ECMO = Veno-Arterial Extracorporeal Membrane Oxygenation; aPTT = activated Partial Thromboplastin Time


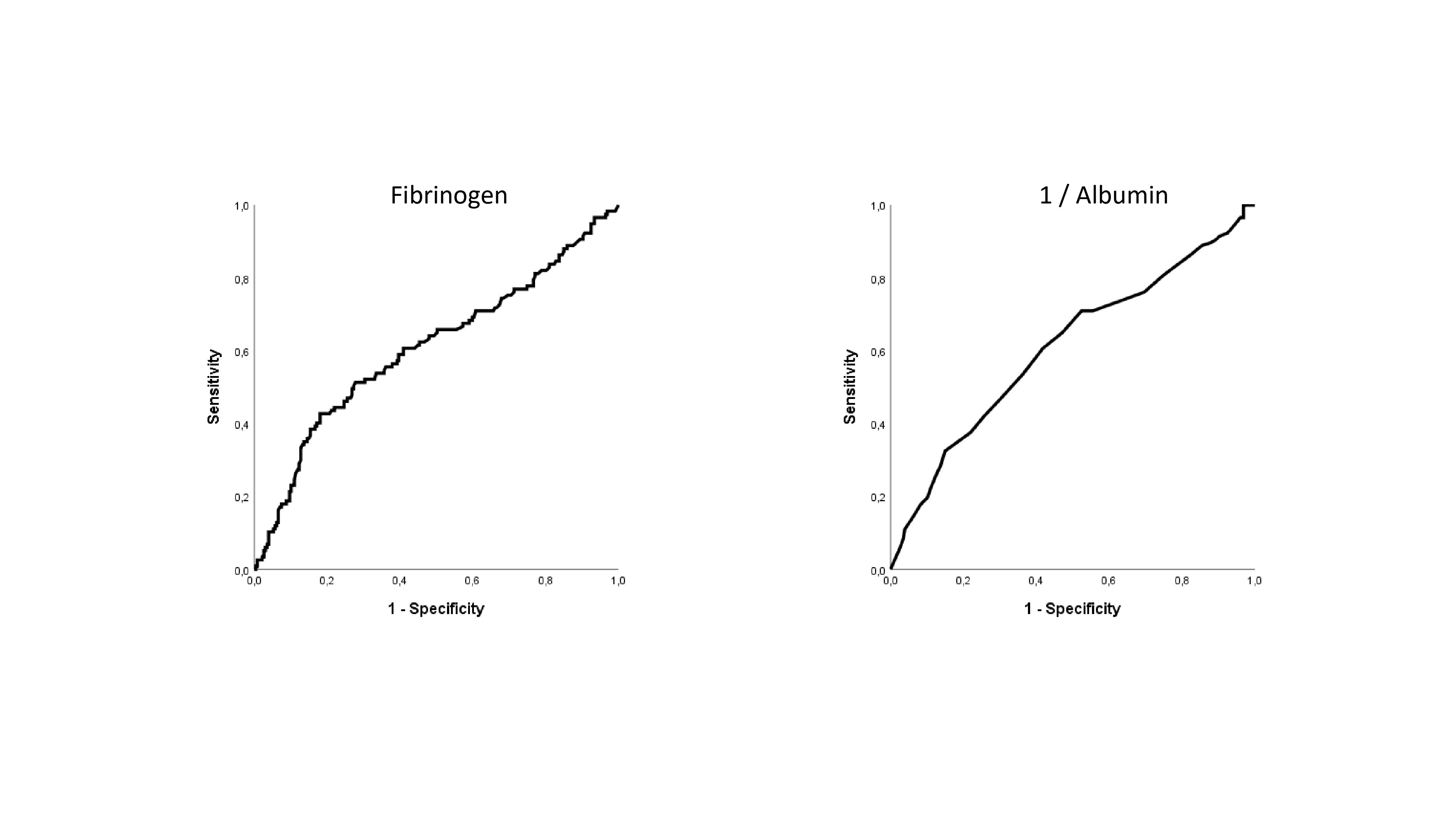


**Supplementary figure 1:** ROC analysis for baseline Fibrinogen and baseline Albumin revealed an AUC of 0.61 [95% CI 0.54-0.67] and 0.61 [95% CI 0.54-0.67], respectively.
